# Supplementary material for: The causal role of circulating inflammatory markers in osteoporosis: a bidirectional Mendelian randomized study
Source: Front Immunol. 2024 Jul 18;15:1412298. doi: 10.3389/fimmu.2024.1412298 (PMC11291241; doi:10.3389/fimmu.2024.1412298)
Supplement: Supplementary file 1 [file DataSheet_1.zip › supplementary material/CIM-OSTEOPOROSIS /GCST90274797.csv_forest.pdf]

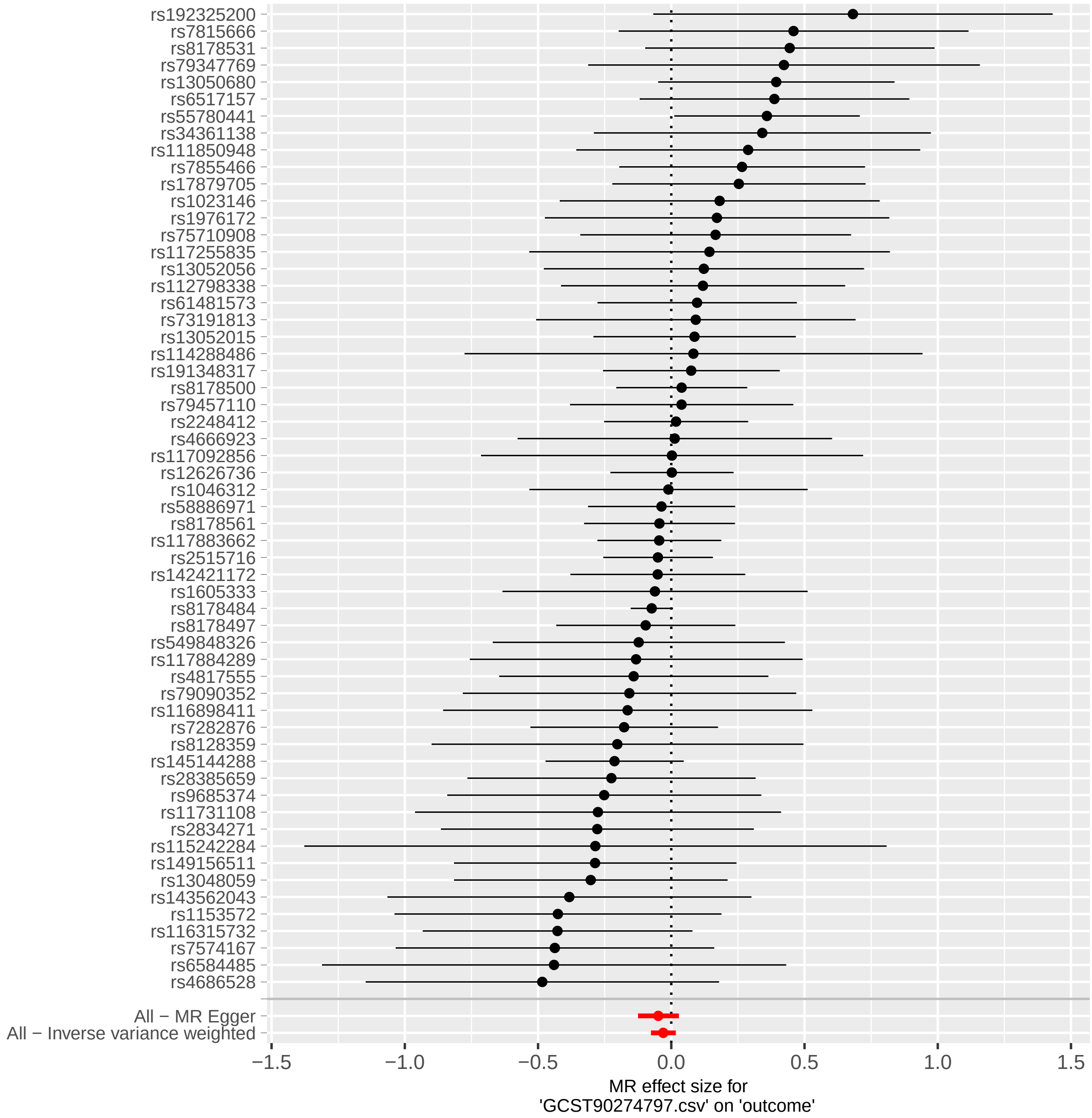
rs192325200
rs7815666
rs8178531
rs79347769
rs13050680
rs6517157
rs55780441
rs34361138
rs111850948
rs7855466
rs17879705
rs1023146
rs1976172
rs75710908
rs117255835
rs13052056
rs112798338
rs61481573
rs73191813
rs13052015
rs114288486
rs191348317
rs8178500
rs79457110
rs2248412
rs4666923
rs117092856
rs12626736
rs1046312
rs58886971
rs8178561
rs117883662
rs2515716
rs142421172
rs1605333
rs8178484
rs8178497
rs549848326
rs117884289
rs4817555
rs79090352
rs116898411
rs7282876
rs8128359
rs145144288
rs28385659
rs9685374
rs11731108
rs2834271
rs115242284
rs149156511
rs13048059
rs143562043
rs1153572
rs116315732
rs7574167
rs6584485
rs4686528
All − MR Egger
All − Inverse variance weighted
−1.5
−1.0
−0.5
0.0
0.5
1.0
1.5
MR effect size for
'GCST90274797.csv' on 'outcome'
